# Mortality, sarcopenic obesity, and sarcopenia: Frailty in Brazilian Older People Study – FIBRA – RJ

**DOI:** 10.11606/s1518-8787.2021055002853

**Published:** 2021-11-08

**Authors:** Glaucia Cristina de Campos, Roberto Alves Lourenço, Maria del Carmen Bisi Molina

**Affiliations:** I Universidade Federal do Espírito Santo Centro de Ciências da Saúde Programa de Pós-Graduação em Saúde Coletiva VitóriaES Brasil Universidade Federal do Espírito Santo. Centro de Ciências da Saúde. Programa de Pós-Graduação em Saúde Coletiva. Vitória, ES, Brasil; II Universidade do Estado do Rio de Janeiro Faculdade de Ciências Médicas Departamento de Medicina Interna Rio de JaneiroRJ Brasil Universidade do Estado do Rio de Janeiro. Faculdade de Ciências Médicas. Departamento de Medicina Interna. Rio de Janeiro, RJ, Brasil

**Keywords:** Aged, Sarcopenia, epidemiology, Obesity, Sarcopenic Obesity, Risk Factors, Mortality

## Abstract

**OBJECTIVE:**

To investigate the risk of mortality associated with sarcopenic obesity (SO), obesity (OB), and sarcopenia in elderlies.

**METHODS:**

We analyzed longitudinal data from 270 participants > 65 years of age of Phase III of the Study on Frailty in Brazilian Older People (FIBRA–RJ–2012). Socioeconomic, demographic, lifestyle, morbidity, and functional data were collected by home based interviews. DXA and body composition assessment was conducted in a laboratory. In women, OB was diagnosed when body fat percentage ≥ 38% and sarcopenia by an Appendicular Lean Mass Index (ALMI) < 6.00 kg/m^2^ and muscle strength < 16 Kgf. In men, OB was diagnosed when body fat percentage ≥ 27%, and sarcopenia was diagnosed with ALMI < 7.00 kg/m^2^ and muscle strength < 27 Kgf. SO was assessed by combining variables used to diagnose obesity and sarcopenia. The probabilistic linkage method was used to obtain deaths in the 2012-January 2017 period from the Brazilian Mortality Registry. Cox regression models were tested, and crude and adjusted hazard ratio calculations were conducted.

**RESULTS:**

After adjusting for sex, age, race/skin color, walking as an exercise, and hypertension, individuals with sarcopenia were 5.7 times more likely to die (95%CI: 1.17–27.99) than others without sarcopenia and obesity.

**CONCLUSION:**

A high risk of death was observed in individuals with sarcopenia. These results show the need for preventive strategies of early detection and treatment in order to increase survival employing multimodal interventions.

## INTRODUCTION

Aging is marked by progressive body composition changes, such as reduced quantity and quality of skeletal muscle mass and increased body fat, which may trigger sarcopenic obesity. There is no consensus in the literature regarding the definition of Sarcopenic Obesity (SO). However, in some studies, it has been characterized as increased fat mass accompanied by reduced muscle mass and muscle strength, i.e., the combination of two body composition phenotypes: sarcopenia and obesity^1,2^.

Studies have emphasized an increasing prevalence of obesity and SO in older adults, with a significant impact on mortality, especially when both these phenotypes are involved^3,4^. However, this association remains unclear due to studies’ diverse designs, cutoff points, assessment methods, and diagnostic criteria^5,6^.

In the United States, Batsis et al.^3^ investigated the association between sarcopenic obesity, sarcopenia, and mortality, and found that only older women with sarcopenia had an increased risk of all-cause mortality, regardless of obesity.

In a longitudinal study with a sample of 4,252 older adults in England, Atkins et al.^6^ showed that, compared to eutrophy, sarcopenic obesity entailed a 1.72 relative risk of all-cause mortality. In another review, Tian & Xu.^4^ analyzed data from prospective cohort studies and showed that individuals with SO have a 24% higher risk of all-cause mortality than those without SO.

While different methods have been used to assess body composition, the multiple compartment model is considered the gold standard in scientific practice for assessing the body composition of individuals with obesity. This method employs a combination of several accurate tools to conduct the anthropometric fractionation of body mass. However, Dual Energy X-Ray Absorptiometry (DXA), a non-invasive technique, has adequate validity, reproducibility, accuracy and is less influenced by body water than other methods^7^.

In low and middle-income countries, few studies use accurate methods to assess aged individuals’ body composition and investigate the risk of death. Thus, this study aims to investigate the risk of mortality associated with sarcopenic obesity, obesity, and sarcopenia in an elderly cohort.

## METHODS

### Study Design, Context, and Sampling

Data from the Rio de Janeiro section of the Cohort Study Fragilidade em Idosos Brasileiros (FIBRA-RJ – Frailty in Brazilian Older People from the state of Rio de Janeiro) was analyzed. The source population of FIBRA is the registered clientele of a health care plan with broad coverage in the State of Rio de Janeiro. The study included those costumers who, in 2009, had been enrolled for at least one year in the plan, aged 65 years or older, and living in the North region of Rio de Janeiro in the study, totaling 847participants.

The sampling was stratified according to sex and age group (65–74 years; 75–84 years, 85–94 years), with data from the healthcare provider’s registry. All participants 95 years or more were included. An inverse sampling strategy was applied to reach the representative sample size for each stratum, and in this case it was not necessary to increase the calculated sample size to compensate for the nonresponse rate. The estimaded sample was based on the prevalence of frailty syndrome, calculated at 12%.The complete methodology of all phases of the FIBRA study is publicly available (10).

Our study analyzed data from the third follow-up wave (FIBRA-RJ - Phase III - 2012/2013), in which body composition was estimated using DXA and mortality data after four years of follow-up in 2017.

In phase III, 136 of the 847 enrolled participants died, and 102 were excluded due to Mini-Mental State Examination result ≤ 13; being institutionalized; wheelchair-bound or bedridden; having difficulty walking; severe hearing and visual impairments that prevented them from answering the questionnaire, and presenting an active neurological or psychiatric disorder. Thus, 609 individuals were eligible. Among these, 64 could not be located, and 143 refused to participate, resulting in a sample of 402 participants who were interviewed at their homes.

**Figure 1:**
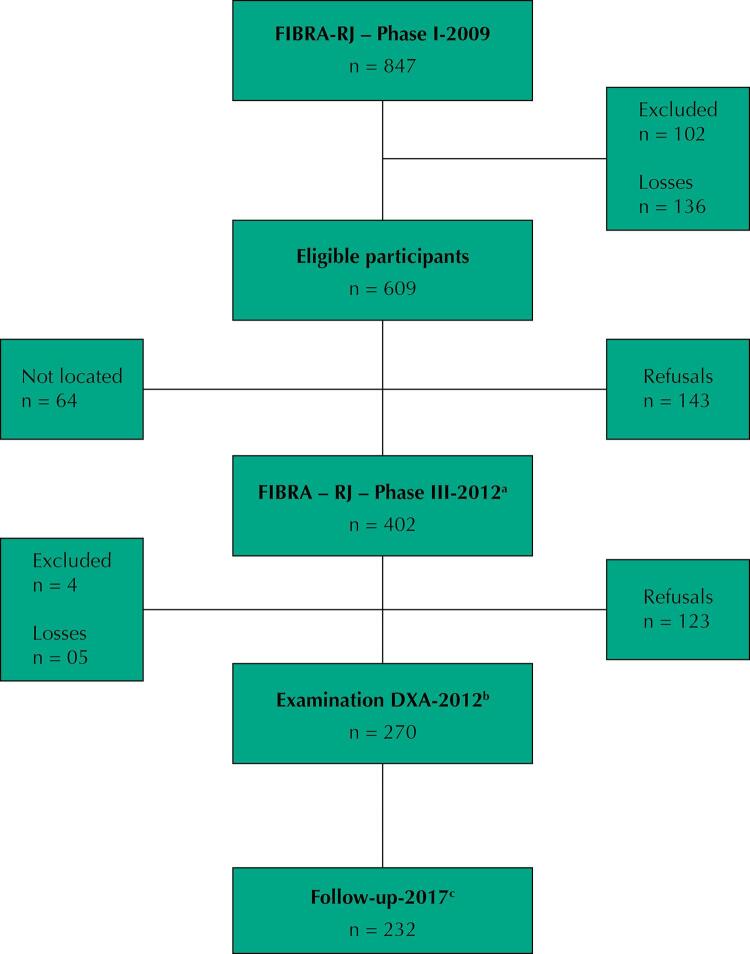
Flowchart diagram for selecting the final sample of the FIBRA RJ-study

^a^ Interview conducted by GERONLAB researchers at the elderly’s residence to conduct the face-to-face interview and to measure anthropometric measurements.

^b^ Attendance of the elderly person to the Interdisciplinary Nutritional Assessment Laboratory to assess body composition through DXA

^c^ Follow-up 2012–2017: 38 deaths.

All respondents were invited to attend the Interdisciplinary Nutritional Assessment Laboratory to assess their body composition, and 270 underwent DXA examination. These constituted the study’s final sample (Figure 1).

### Mortality

The probabilistic linkage method was used to obtain deaths in the 2012-January 2017 period from the Brazilian Mortality Registry, which led to the identification of 38 post-phase III (2012) deaths. Two variables were ascertained: outcome and date of death (month/day/year). Follow-up time was calculated in months and years from the interview date to the date of death, or from the most recent vital status record. Mortality data were complete in 100% of the sample.

### Measures

#### Anthropometric measurements, body composition, and muscle-strength

The individuals were submitted to nutritional assessment using the FIBRA RJ-III research protocol. During scheduling, all participants received previous guidance on how to prepare for the tests.

All participants had their anthropometric measurements (weight and height) gauged using the methods described by Lohman et al.^10^. We used a tape measure affixed to the wall to measure height, with individuals standing barefoot. The numbers were recorded in millimeters, without any rounding. Weight was obtained using a portable Plenna/Everest TIN-00110 microelectronic scale with 150kg capacity and 200g accuracy. For weight measurement, the barefoot participants wore light clothes. Weight measurements were recorded in tenths of a kilogram, without rounding.

To determine bone and tissue mineral density, DXA (Lunar iDXA model, General Electric) was used to assess body composition. Tissues are fractionated into fat mass and fat-free mass. The upper and lower limbs are isolated from the trunk and head by program-generated lines; measurements related to these body parts can then be manually adjusted with a high degree of accuracy. Appendicular muscle mass was characterized using the appendicular lean mass index (ALMI), obtained by calculating the sum of arms’ and legs’ lean masses (lean tissue [kg] + bone mineral content [kg]) divided by height squared (2).

Muscle strength was measured using a three-replicate protocol with regular time intervals. To this end, a Jamar hydraulic hand dynamometer (NC 701/42 – North Coast) was used to assess strength in the dominant hand. The device’s strap was adjusted to the size of the individual’s hand, using position II as a point of reference. The respondent was asked to exert the highest possible force, and the three measurements were performed. Their mean was subsequently calculated^2^.

#### Sarcopenia

Females with muscle strength < 16 Kgf and ALMI < 6.0 kg/m^2^, and males with muscle strength < 27 Kgf and ALMI < 7.0 kg/m^2^, were considered to have sarcopenia.

#### Sarcopenic obesity

SO was assessed by combining variables adopted for diagnosing sarcopenia with variables used for diagnosing obesity. Females were considered to have sarcopenic obesity when their body fat percentage was ≥ 38%, their ALMI < 6.0 kg/m^2^, and muscle strength < 16 Kgf. Males were considered to have sarcopenic obesity when their body fat percentage was ≥ 27%, their ALMI < 7.0 kg/m^2^, and muscle strength < 27 Kgf. (2).

## Covariates

Socioeconomic and demographic variables included the following variables: age, sex, race/skin color, and income. Age was measured in full years and separated into two groups (< 75 years; ≥ 75 and over). Participants were classified as retirees, pensioners, or as counting on other sources of personal income. Individual income from work, retirement, or pension was assessed in minimum wages (MW) at the time of the interview. Participants were thus categorized as follows: 0 to 2 (MW) - 0 to 668.62 (USD), 2.1 to 5 (MW) -668.63 to 1,671.55 (USD), and > 5 (MW) - > 1,671.56 (USD). Race/skin color was categorized as either black/ brown and white.

Lifestyle-related variables analyzed were: tobacco use (currently smoking, never smoked/quit smoking) and walking as an exercise (yes/no).

The following physical morbidities were investigated: diabetes mellitus, hypertension, and heart disease. To this end, the question ‘Has any doctor ever said that you have the following health problems?’ (diabetes mellitus [yes/no], hypertension [yes/no], and heart disease [yes/no]) was asked.

## Statistical Analysis

The descriptive analysis was conducted by frequency distribution for categorical variables and then Pearson’s chi-square test was applied. The body composition phenotypes exposure variable was categorized into four groups: absence of sarcopenia and without obesity, sarcopenia, obesity, and sarcopenic obesity, and the risk of death was estimated using Cox proportional-hazards regression model.

The Kaplan–Meier estimator and the log-rank test were used to assess the proportionality of survival curves, which were base for estimation of hazard ratios (HR) by the Cox regression model. For Cox regression, variables with *p-value* < 0.20 in the bivariate analyses were included in the model as covariates. Mortality-related HRs were initially calculated without any adjustment. Model 1 was adjusted for sociodemographic variables (sex, age and race/skin color); model 2 for sociodemographic variables + lifestyle (walking as exercise); and model 3 for sociodemographic variables + lifestyle + morbidity (Hypertension). Given the study’s design, statistical analysis was performed using the statistical package SPSS for Windows version 22.

## Ethical Aspects

The selected individuals who consented to the home visit only answered the questionnaire after signing an Informed Consent Form. The original research followed the recommendations of Resolution 196/96 of the National Health Council of the Ministry of Health. In the first phase of the FIBRA study, it was approved by the Research Ethics Committee of the Pedro Ernesto University Hospital (2007, opinion number 1850), and the National Research Ethics Committee (CONEP) (2012, opinion number 120.700) in the third phase.

## RESULTS

Two hundred seventy (270) individuals with a mean age of 77.5 years (SD = ± 5.92), predominantly females (70%), were analyzed. Thirty-eight participants (14.1%) died during the 4-year follow-up. Table 1 shows the prevalence of obesity, sarcopenic obesity, and sarcopenia, according to sociodemographic and economic characteristics, lifestyle, and morbidities. Depending on sex and age group, statistically significant differences between individuals with sarcopenic obesity, obesity, sarcopenia, and the absence of sarcopenia and without obesity.

**Table 1:** Frequency of body composition phenotypes according to socio demographic and economic characteristics, morbidities, and lifestyle in elderlies (FIBRA III, RJ, Brazil-2012)

Variables		Eutrophy^a^	Sarcopenia	Obesity	Sarcopenic Obesity	p
			
		n (%)	n (%)	n (%)	n (%)	
Sex	Female	36 (19.0)	15 (7.9)	113 (59.8)	25 (13.2)^a^	0,00
	Male	9 (11.1)	11 (13.6)	34 (42.0)	27 (33.0)	-
Race/Skin color	White	24 (14.8)	14 (8.6)	85 (52.5)	39 (24.1)	0.09
	Black/Brown	21 (19.4)	12 (11.1)	62 (57.4)	12 (13)	-
Income in MW^b^	0 to 2	4 (8.2)	4 (8.2)	29 (59.2)	12 (24.5)	
	2.1 to 5	21 (17.6)	11 (9.2)	67 (56.3)	20 (16.8)	0,47
	> 5	19 (21.0)	9 (10.2)	42 (47.7)	18 (20.5)	-
Age Group	65 to 74.9	17 (16.8)	6 (5.9)	65 (64.4)	13 (12.9)*	0,03
	≥ 75	28 (16.6)	20 (11.8)	82 (48.5)	39 (23.1)	-
Diabetes Mellitus	No	36 (18.1)	18 (9)	108 (54.3)	37 (18.6)	0,75
	Yes	9 (13.0)	8 (11.6)	38 (55.1)	14 (20.3)	-
Arterial Hypertension	No	18 (18.2)	12 (12.1)	45 (45.5)	24 (24.2)	0,12
	Yes	27 (16.0)	14 (8.3)	101 (59.8)	27 (16.0)	-
Heart disease	No	34 (16.4)	19 (9.2)	117 (55.5)	37 (17.9)	0,64
	Yes	11 (18.0)	7 (11.5)	29 (47.5)	14 (23.0)	
Smoking	No	25 (15.5)	18 (11.2)	91 (56.5)	27 (16.8)	0,67
	Yes	3 (25.0)	0 (0.0)	6 (50.0)	3 (25.0).	-
	Ex-smoker	17 (17.5)	8 (8.2)	50 (51.5)	22 (22.7)	
Walking	No	34 (16.5)	17 (8.3)	114 (55.3)	41 (19.9)	0,33
	Yes	11 (17.2)	9 (14.1)	33 (51.6)	11 (17.2)	-

^a^ Eutrophy = Without sarcopenia and obesity.

^b^ MW = Minimum Wage in 2012 ($ 334,31).

We identified a higher prevalence of sarcopenic obesity and sarcopenia among males (33% and 13.6%, respectively); paradoxically, the prevalence of obesity in females was higher (59.8%) and statistically significant. The prevalence of sarcopenic obesity in individuals under 75 years of age was lower (12.9%), but higher in obese subjects (69%).

The prevalence of diabetes mellitus was higher in obese (55.1%) than sarcopenic obese individuals (20.3%), but without statistical significance. High proportions of obesity (59.8%) and sarcopenic obesity (16%) were observed in

individuals with hypertension, again without statistically significant difference. Regarding lifestyle-related variables, a high prevalence of sarcopenic obesity and obesity was identified in individuals who had stopped smoking (51.5% and 22.7%, respectively). There were also high proportions of sarcopenic obesity (17%) and obesity (51.6%) among individuals who did not practice walking as an exercise.

Females had higher mean fat percentage and mean appendicular lean mass index values when compared to males (Table 2).

**Table 2:** Baseline Anthropometric and body composition characteristics of participants.

Variables	Female	Male	Total	p
		
	Mean ± SD	Mean ± SD	Mean ± SD	
Body Mass Index (kg/m^2^)	28 ± 7.83	28.1 ± 12.13	28.0 ± 9.2	0.93
Fat Mass (kg)	27.0 ± 9.0	22.6 ± 6.93	25.7 ± 8.6	< 0.0001
Lean Mass (kg)	35.6 ± 5.1	45.9 ± 5.87	38.6 ± 7.1	< 0.0001
Total Mass (kg)	64.5 ± 13.2	71.0 ± 11.06	66.4 ±12.9	< 0.0001
ALMI (kg/m^2^)	6.2 ± 2.0	4.7 ± 1.46	5.8 ± 2.0	< 0.0001
Fat percentage (%)	41 ± 6.5	31.3 ± 6.2	38.1 ± 7.8	< 0.0001
Hand grip force (kgf)	17.6 ± 5.1	26.8 ± 6.47	20.3 ± 6.9	< 0.0001

ALMI: appendicular lean mass index.

**Figure 2:**
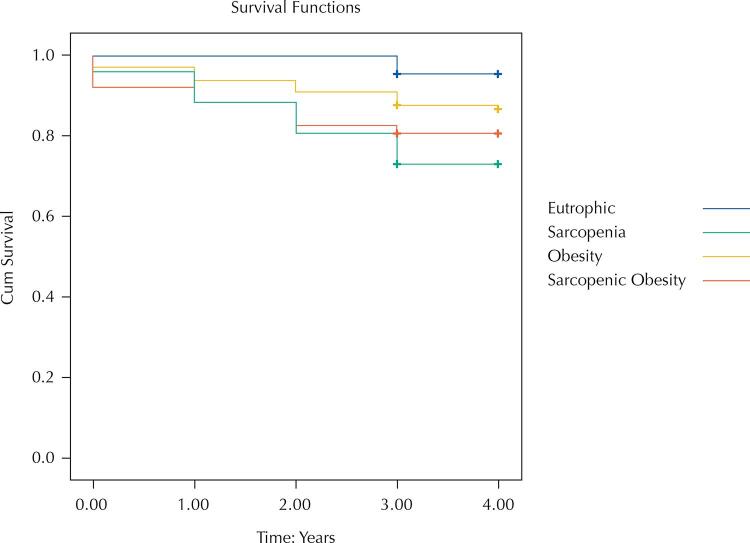
Kaplan–Meier survival curves according to body composition phenotypes in elderlies (FIBRA-RJ, Brazil, 2012–2017).

Figure 2 shows that Kaplan–Meier survival curves were proportional and differed statistically among sarcopenic obese, obese, sarcopenic, and eutrophic individuals (absence of sarcopenia and without obesity). Individuals with sarcopenia, sarcopenic obesity, and obesity survive less than the absence of sarcopenia and without obesity.

Log Rank = 0.03

Table 3 describes the crude and adjusted risk of death HRs according to body composition phenotypes. Crude analyses show that the following variables led to a statistically significant higher risk of death: sarcopenia HR = 6.22 (95% CI 1.29–29.96) and SO HR = 4.67 (95%CI: 1.02–21.35). In the final model, in which the analysis was adjusted to sociodemographic variables (sex, age, and race/skin color), lifestyle (walking as an exercise), and hypertension, sarcopenic individuals were 5.73 (95%CI: 1.17–27.99) more likely to be at risk of death than with the absence of sarcopenia and without obesity, and the difference was statistically significant.

**Table 3:** Associations between body composition phenotypes and mortality in elderlies (FIBRA-RJ, Brazil, 2012–2017).

Body Composition Phenotypes	Crude HR (95%CI)	Model 1^a^	Model 2^b^	Final Model^c^
		
		Crude HR (95%CI)	Adjusted HR (95%CI)	Adjusted HR (95%CI)
Eutrophy	1	1	1	1
Sarcopenia	6.22 (1.29–29.96)	5.17 (1.06–25.13)	5.42 (1.11-26.40)	5.73 (1.17–27.99)
Obesity	2.96 (0.69–12.74)	3.04 (0.70–1.11)	3.01 (0.71–1.3)	2.84 (0.65–12.30)
Sarcopenic Obesity	4.67 (1.02–21.35)	3.80 (0.81–17.73)	3.70 (0.79–17.19)	3.74 (0.80–17.49)

^a^ Model 1 – Body composition phenotypes adjusted for sociodemographic variables (sex, age and race/color).

^b^ Model 2 – Body composition phenotypes adjusted for sociodemographic variables and lifestyle variable (walking as exercise).

^c^ Model 3 – Final Model-Body composition phenotypes adjusted for sociodemographic variables, lifestyle variable (walking as exercise), and morbidity (systemic arterial hypertension).

## DISCUSSION

In this Brazilian study, sarcopenic obesity and obesity were associated with a higher risk of death, although the difference was not statistically significant. Older adults with sarcopenia were five times more likely to be at risk of mortality than with the absence of sarcopenia and without obesity individuals. These results corroborate international studies, such as those by Batsis and Villareal^1^, Kim et al.^11^, Liu et al.^12^, and Sanada et al.^13^.

Kim et al.^11^ analyzed data from 556 older adults, participants of the Korean Longitudinal Study on Health and Aging (KLoSHA). Sarcopenic men were 5.37 times more likely to be at risk of death. A meta-analysis performed by Liu et al.^12^ between 2009 and 2017 – which included six prospective cohort studies incorporating 7367 participants – also identified sarcopenia as a predictor of all-cause mortality. Sarcopenic individuals had significantly higher risk ratios than non-sarcopenic ones. In a study by Sanada et al.^13^ involving 2,309 Japanese older adults in the United States, the authors observed that the risk of death from all causes was higher in seniors with sarcopenic obesity (HR = 1.19; 95%CI: 1.02–1.38) and sarcopenia (HR = 1.26; 95%CI: 1.15–1.38) than their peers without obesity and sarcopenia.

One of the possible reasons for the higher risk of death in sarcopenic older adults is related to adverse outcomes. Sarcopenia has been widely investigated as one of the main risk factors for functional dependence, falls, frailty, institutionalization, and death^1,3,14^. Moreover, regardless of how sarcopenia is diagnostically defined, low muscle mass and low muscle strength are important risk factors for disability and increased mortality^15,16^. In this sense, the prevention of sarcopenia should be one of the main objectives of public health professionals.

While comparing sarcopenic and non-sarcopenic individuals, Beaudart et al.^17^ observed that sarcopenic individuals consumed a more significant mean number of routine medicines, had a higher number of comorbidities, respiratory problems, kidney problems, hospitalizations in the year before the visit, and suffered from malnutrition or risk of malnutrition more often. They also had a lower cognitive capacity, worse health-related physical quality of life, higher risk of falls, were more fragile, and got more tired when performing daily activities. Furthermore, seniors with sarcopenia have a lower response rate to treating acute diseases and worse healing of wounds and fractures, leading to worse prognosis^18,19^.

Our study found a high prevalence of obesity and SO. Both were associated with a higher risk of death, although the difference was not statistically significant. There were statistically significant higher proportions of obesity in younger (< 75 years) and women. The prevalence of sarcopenic obesity and obesity among older adults in Brazil and worldwide has increased in recent years^20^. In this population, obesity can cause changes in body composition and muscle quality and is linked to increased incidence of sarcopenia, increased risk of nutritional deficiencies, lower quality of life, disability, frailty, and shorter lifespan^24^.

The survival bias effect may explain the declining prevalence of obesity in more advanced ages. The negative impact of obesity and its associated morbidities contributes to the higher mortality observed in obese individuals below 80 years of age^25^. On the other hand, one possible explanation for the higher prevalence of obesity in women may be their higher visceral fat accumulation and longer life expectancy^26^.

The proportion of participants with SO was high, corroborating other studies^22,27^. Sarcopenic obesity was at risk of death but without statistical significance. In this sense, some international studies have shown conflicting results with significant associations between sarcopenic obesity and death risk^4^. Some discrepancies were identified in the proportions and results determined, probably due to the sample size and the different criteria and methods used to assess the risk of death. In a literature review, in a meta-analysis, Tian & Xu^4^ analyzed data from nine articles with 12 prospective cohort studies and showed that individuals with SO are 24% more likely to be at risk of all-cause mortality than those without SO. However, they observed conflicting results with four studies showing significant associations and four not showing statistical significance.

In the United States, Van Aller et al.^28^ analyzed longitudinal data from 3,577 participants of the National Health and Nutrition Examination Survey III (1999–2004) aged 50 years or over. They found that SO increased the risk of mortality in people aged 50 to 70 years, but not in people aged 70 years or over. Both obesity and sarcopenia are associated with metabolic disorders and are important causes of disability, morbidity, and mortality^1,24,29^. Studies with conflicting results strengthen consensus building and contribute to the development of more precise methods for the diagnosis of SO. Due to the lack of standardization of criteria and methods for SO diagnosis, its impact on mortality remains unclear^18^.

Considering this study’s results, we reinforce the need for interdisciplinary planning of preventive and therapeutic actions targeting sarcopenia and SO. These actions must contemplate multimodal care via the promotion of healthy eating, hyperproteic diets and vitamin D, associated with aerobic physical activity^30^. Health professionals are increasingly able to prevent, delay, treat, and sometimes even reverse sarcopenia through early and effective interventions^2^.

Our study had some limitations. The lack of consensus and cut-off points validated for the Brazilian population is one of them. The small sample size in phase III, losses during follow-up, and the FIBRA study’s closed cohort. Also, representativeness is limited, as our sample consisted of individuals with health insurance and, therefore, with better access to health services and a higher socioeconomic level than those in the population of Rio de Janeiro in general. Another limitation may be related to possible information bias because chronic diseases were self-reported, influencing our estimates. Regarding the strengths of our research, we highlight that, to the best of our knowledge, it is the first Brazilian longitudinal study employing a more accurate method (DXA) to investigate mortality data in older adults with sarcopenia, obesity, and sarcopenic obesity. It can thus be inferred that the associations found are also more robust. Thus, to the best of our knowledge, our study appears to be pioneering the issue in Brazil. In addition, we conduct training sessions, develop protocols for the interviewers before the collection of the study and quality control throughout the collection.

## CONCLUSION

In this cohort, a high risk of death was observed in individuals with sarcopenia, reinforcing the need for actions to prevent and treat this muscular disease. A high, albeit not significant risk was also found among participants with SO and obesity. These results show the need for multimodal policies to promote healthy eating and physical activity, increasing these individuals’ survival and preventing sarcopenia. Guidelines to promote healthy aging should be directed maintenance or increased muscle mass to boost survival.
